# Diagnostic Strategies for Postmenopausal Bleeding

**DOI:** 10.1155/2010/850812

**Published:** 2010-02-04

**Authors:** M. C. Breijer, A. Timmermans, H. C. van Doorn, B. W. J. Mol, B. C. Opmeer

**Affiliations:** ^1^Department of Clinical Epidemiology, Biostatistics and Bioinformatics, Academic Medical Center, University of Amsterdam, Meibergdreef 9, 1105 AZ Amsterdam, The Netherlands; ^2^Department Obstetrics and Gynecology, TweeSteden Hospital, Dr. Deelenlaan 5, 5042 AD Tilburg, The Netherlands; ^3^Department Obstetrics and Gynecology, Academic Medical Center, Amsterdam, The Netherlands; ^4^Department Obstetrics and Gynecology, Erasmus MC, s-Gravendijkwal 230, 3015 CE Rotterdam, The Netherlands; ^5^Department Obstetrics and Gynecology, Maxima Medisch Centrum, De Run 4600, 5504 DB Veldhoven, The Netherlands

## Abstract

Postmenopausal bleeding (PMB) is a common clinical problem. Patients with PMB have 10%–15% chance of having endometrial carcinoma and therefore the diagnostic workup is aimed at excluding malignancy. Patient characteristics can alter the probability of having endometrial carcinoma in patients with PMB; in certain groups of patients the incidence has been reported to be as high as 29%. Transvaginal sonography (TVS) is used as a first step in the diagnostic workup, but different authors have come to different conclusions assessing the accuracy of TVS for excluding endometrial carcinoma. Diagnostic procedures obtaining material for histological assessment (e.g., dilatation and curettage, hysteroscopy, and endometrial biopsy) can be more accurate but are also more invasive. The best diagnostic strategy for diagnosing endometrial carcinoma in patients with PMB still remains controversial. Future research should be focussed on achieving a higher accuracy of different diagnostic strategies.

## 1. Introduction

Postmenopausal bleeding (PMB) can be defined as uterine bleeding occurring at least one year after menopause. PMB is a common clinical problem in both general and hospital settings [1, 2]. The incidence of spontaneously occurring PMB in the general population can be as high as 10% immediately after menopause [3]. 

PMB is often caused by abnormalities of the endometrium, whether they are benign or malignant. Of postmenopausal women with vaginal bleeding, 10%–15% have endometrial carcinoma [4–8]. In contrast, the prevalence of endometrial polyps in patients with PMB and an increased endometrial thickness measured with transvaginal sonography (TVS) is estimated to be around 40% [9, 10].

Endometrial cancer is the most common malignancy of the female genital tract in developed countries [11]. Unlike other malignancies, endometrial cancer often presents at an early stage when there is a possibility of curative treatment by hysterectomy. Survival decreases with increased staging and lower histological differentiation, thus accurate and timely diagnosis is important and should preferably be carried out by a safe, simple and minimally invasive method. Guidelines addressing PMB are therefore aimed at excluding cervical cancer, endometrial carcinoma or precancerous lesions of the endometrium [12–15].

## 2. Diagnosis of Endometrial Carcinoma

### 2.1. Accuracy of Transvaginal Ultrasonography for Diagnosing Endometrial Carcinoma

Since two decades TVS has become widely used in the evaluation of women with PMB. Before TVS was introduced in the early 1990s, women with PMB were scheduled for dilatation and curettage (D&C). The goal of TVS assessment of the endometrium is to exclude endometrial carcinoma. The probability of endometrial pathology is strongly reduced in the presence of an endometrial ultrasound with an endometrial thickness ≤4 mm. Endometrial sampling is not recommended below this cutoff value [16–18]. 

Guidelines [12–15] almost always refer to a meta-analysis performed by Smith-Bindman et al. [17]. Although this is the most cited publication, there are three meta-analyses on this subject which have used different methods and have come to different conclusions [16–18].

The meta-analysis of Smith-Bindman et al. [17] combined published data from different studies. Using the reported data, 2 × 2 tables per included study were constructed that compared endometrial thickness measured at TVS to presence or absence of endometrial carcinoma. Results across studies were combined in a summary Receiver Operator Characteristics (ROC) Curve. At a 5 mm cutoff the sensitivity for detecting endometrial cancer was 96% for a 39% false-positive rate. Such a combination of sensitivity and specificity would reduce a pretest probability of 10% for endometrial cancer to a posttest probability of 1% [17]. Based on this posttest probability, expectant management is at present recommended to these women.

Gupta et al. [16] conducted a comprehensive systematic review in which they focused on the study quality assessment of each study. Only four studies were identified as best-quality studies [19–22]. For each paper a 2 × 2 table was constructed and likelihood ratios (LR) were calculated. Pooling of the results of these four studies for endometrial thickness ≤5 mm resulted in a LR of a negative test of 0.16. In a patient with a negative test result, the posttest probability was 2.5% [16].

Tabor et al. [18] included only studies from which they were able to get the original data from the authors. For each study they calculated median endometrial thickness per centre and used multiples of the median for endometrial thickness to pool data. They reported a sensitivity of 96% for a specificity of 50% and concluded that such a sensitivity with a 4% false-negative rate was too high. Therefore, in their opinion endometrial thickness measurement does not reduce the need for invasive diagnostic testing [18].

Besides the test accuracy, the pretest probability (before any test is done) influences the performance of a diagnostic test in clinical practice. The pretest probability is approximately 10% for the whole population of patients with PMB, but various clinical characteristics can alter this pretest probability. The probability of endometrial carcinoma in women with PMB rises from 1% in women younger than 50 years to 23.8% in women older than 80 years and the incidence of malignancy is, regardless of age, higher in women with PMB and obesity (18%) or diabetes (21%) as compared to women without one of these risk factors (8.0%) [23]. In obese women with diabetes the incidence is reported to be as high as 29% [23]. As the pretest probability for malignancy is higher, the potential of the test to reduce the posttest probability to below 5% can be limited.

### 2.2. Accuracy of Invasive Endometrial Assessment Methods

Patients with an increased endometrial thickness should undergo more invasive testing, that is, office endometrial sampling, hysteroscopy or dilation and curettage (D&C), to exclude endometrial pathology.

D&C was traditionally the method of choice for investigating patients with postmenopausal bleeding. However, in approximately 60% of the D&C procedures less than half of the uterine cavity is curetted. Another drawback of D&C is that this procedure is performed under general anaesthesia in an inpatient setting [24]. D&C is now considered to be outdated practice and is replaced by less invasive outpatient evaluation using endometrial biopsy devices and outpatient hysteroscopy guided biopsies [25].

Guidelines advocate office endometrial sampling to rule out endometrial carcinoma in women with PMB and an increased endometrial thickness, measured with TVS. Dijkhuizen et al. [26] performed a meta-analysis comparing different minimally invasive endometrial biopsy devices. In postmenopausal women endometrial sampling with both the Pipelle device (Pipelle de Cornier, Paris, France) and the Vabra device (Berkeley Medevices, Inc., Richmond, Calif, USA) are very sensitive techniques for the detection of endometrial carcinoma, with detection rates of 99.6% and 97.1%, respectively, [26]. Despite these reassuring features, the amount of tissue obtained by office sampling varies considerably and is sometimes insufficient for a reliable histological diagnosis. In case the material is classified as insufficient, the clinician is in doubt whether or not to proceed with more invasive testing or to rely on the negative biopsy. In a prospective study performed by Van Doorn et al. four (6%) out of 66 patients with insufficient tissue at office endometrial sample were subsequently diagnosed with endometrial cancer (*n* = 3) or atypical hyperplasia (*n* = 1). This finding implicates that women with an insufficient sample and an endometrial thickness of 5 mm or more should not be reassured [27].

Compared with traditional methods such as curettage, hysteroscopy offers the possibility of visualizing macroscopic or focal abnormalities and taking directed biopsies [28, 29]. With the development of smaller diameter hysteroscopic systems and the introduction of a “vaginoscopic” approach to hysteroscopy (without the use of a speculum or tenaculum), patient acceptance has improved considerably and hysteroscopy nowadays can be performed in an outpatient setting without the use of anaesthesia [30, 31].

## 3. Diagnostic Strategies for Postmenopausal Bleeding

In clinical practice, tests are commonly combined in diagnostic sequences and disease probabilities are usually estimated in a hierarchical manner, first combining information from history and patient characteristics followed by information from additional testing. Test accuracy studies often do not take this clinical paradigm into account. They usually report on the status of a test disregarding history and patient characteristics. Assessing tests in isolation of other tests in the diagnostic sequence (including information from clinical history and patient characteristics) exaggerates the diagnostic information that test combinations can provide in practice.

To determine the most cost-effective testing strategy for diagnosing endometrial carcinoma in women with PMB, Clark et al. constructed a decision model and evaluated 12 different strategies for the initial investigation of PMB. Depending on cancer prevalence (5% versus 10%, resp.), a strategy with TVS as initial investigation with a cut-off of 5 mm or 4 mm followed by endometrial biopsy was most cost effective [1].

There is considerable variability in the endometrial thickness and the likelihood of endometrial carcinoma across women. This variability has been associated with individual patient characteristics including age, time since menopause, obesity, hypertension, diabetes mellitus, and reproductive factors [23, 32–36]. However, guidelines currently used are mainly based on endometrial thickness only, and do not systematically take these additional characteristics into account [12–15].

Inclusion of these individual characteristics may allow for a more refined differentiation of women with the same endometrial thickness. This could result in a more individualised and possibly more accurate and efficient work-up strategy, in which a very high a priori chance of endometrial carcinoma warrants further histological testing, whereas women with a very low prior chance might be reassured even without TVS.

Multivariable models to predict endometrial carcinoma incorporating patient characteristics in the diagnostic work-up for patients with PMB have been developed [37–40]. Khan et al. proposed the use of individual patient data meta analysis in developing these multivariable models to calculate a posttest probability of disease for a different combination of test results (including patient characteristics and information from clinical history) [38].

**Figure 1:**
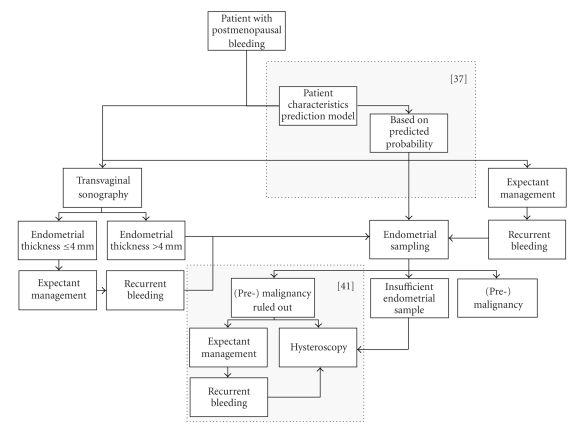
Possible diagnostic pathways for postmenopausal bleeding. The areas surrounded by a dotted square require futher research.

Figure 1 shows an algorithm with *possible* diagnostic pathways for PMB. In this figure an evidence-based approach is combined with approaches requiring more research. Two areas require further research: (1) probability modelling to calculate the pretest probability of endometrial cancer based on patient characteristics [37] and the implementation of such a model in the diagnostic strategy and finally implementation into daily practice and (2) diagnostic approach to benign pathology. That is wether or not subsequent endometrial cavity evaluation for benign abnormalities should be performed after malignancy has been ruled out [41].

## 4. General Conclusions and Future Research

Sensitivity of TVS endometrial thickness measurement in women with PMB is still controversial. Future research should aim at achieving a higher accuracy of the diagnostic strategy applied. Such higher accuracy might be achieved by incorporation of patient's characteristics (e.g., age, presence of diabetes, Body Mass Index (BMI), presence of hypertension) in the diagnostic work-up. The incorporation of TVS with patient's characteristics in a diagnostic strategy has been studied and resulted in higher diagnostic accuracy [37, 39, 40]. Statistical methods can be used to develop and further improve such models and incorporating patient's characteristics with diagnostic tests [38, 40]. Furthermore, by combining and analysing individual patient data from different studies (IPD meta analyses), larger databases can be obtained, in which previously described models can be externally validated [38, 42]. Such models could be incorporated in clinical prediction rules, where the individual probability for endometrial cancer is obtained for each individual woman, and a diagnostic algorithm is developed to maximize the diagnostic accuracy at an acceptable patient burden and health care costs. Such prediction rules are currently also available in reproductive medicine, and comparable to the risk of malignancy index in ovarian tumours [43, 44]. After developing such clinical prediction rules, diagnostic accuracy and clinical applicability should be tested in clinical practice in a prospective multicentre study. If indeed, such model; would lead to higher diagnostic accuracy than TVS alone, office endometrial sampling or office hysteroscopy could then be offered only to those women with a high probability of endometrial cancer and its precursors.
